# Fabrication of Porous Anodic Alumina (PAA) by High-Temperature Pulse-Anodization: Tuning the Optical Characteristics of PAA-Based DBR in the NIR-MIR Region

**DOI:** 10.3390/ma13245622

**Published:** 2020-12-09

**Authors:** Ewelina Białek, Maksymilian Włodarski, Małgorzata Norek

**Affiliations:** 1 Faculty of Advanced Technologies and Chemistry, Institute of Materials Science and Engineering, Military University of Technology, Str. gen Sylwestra Kaliskiego 2, 00-908 Warsaw, Poland; ewelina.bialek2@gmail.com; 2 Institute of Optoelectronics, Military University of Technology, Str. gen. Sylwestra Kaliskiego 2, 00-908 Warsaw, Poland; maksymilian.wlodarski@wat.edu.pl

**Keywords:** porous anodic alumina (PAA), pulse anodization, mid-infrared (MIR), near-infrared (NIR), distributed Bragg reflector (DBR), photonic stop band (PSB)

## Abstract

In this work, the influence of various electrochemical parameters on the production of porous anodic alumina (PAA)-based DBRs (distributed Bragg reflector) during high-temperature-pulse-anodization was studied. It was observed that lowering the temperature from 30 to 27 °C brings about radical changes in the optical performance of the DBRs. The multilayered PAA fabricated at 27 °C did not show optical characteristics typical for DBR. The DBR performance was further tuned at 30 °C. The current recovery (*i_a_^max^*) after application of subsequent U_H_ pulses started to stabilize upon decreasing high (U_H_) and low (U_L_) voltage pulses, which was reflected in a smaller difference between initial and final thickness of alternating d_H_ and d_L_ segments (formed under U_H_ and U_L_, respectively) and a better DBR performance. Shortening U_H_ pulse duration resulted in a progressive shift of photonic stopbands (PSBs) towards the blue part of the spectrum while keeping intensive and symmetric PSBs in the NIR-MIR range. Despite the obvious improvement of the DBR performance by modulation of electrochemical parameters, the problem regarding full control over the homogeneous formation of d_H_+d_L_ pairs remains. Solving this problem will certainly lead to the production of affordable and efficient PAA-based photonic crystals with tunable photonic properties in the NIR-MIR region.

## 1. Introduction

Distributed Bragg reflectors (DBRs) are 1D photonic crystals (PCs) built of alternate low and high refractive index (RI) layers designed to forbid specific light to propagate in the structure. This phenomenon arises from the partial reflection of a wavelength that is close to four times the optical thickness of the layers from each layer boundary. As a consequence of constructive interference of the multiple reflections, DBR demonstrates high reflectance (or low transmittance) of light in a given part of the spectrum, which is called photonic stopband (PSB). DBRs are frequently used in LEDs [1,2], solar cells [3,4], or vertical-cavity surface-emitting lasers [5,6] as important optical components that enhance luminous or light trapping efficacy. Conventional DBRs are made of two different materials that require careful consideration of their optical and mechanical properties, as well as when choosing a reliable/reproducible method to grow the anticipated structure. This approach has many limitations, including thermal expansion mismatch and necessity to use costly equipment. An alternative approach that can avoid these problems relies on the production of the same material-based DBRs. These kinds of photonic structures were frequently built of indium tin oxide (ITO) [7], amorphous silicon (a-Si) [8], amorphous germanium (a-Ge) [9], or titanium dioxide (TiO_2_) [10] by the oblique angle deposition (OAD) technique, also known as the glancing angle deposition (GLAD) [11]. The OAD methods usually employ electron beam evaporation or magnetron sputtering in oblique geometric configuration with rotating substrates. Despite the inevitable attractiveness of the OAD, such as controllability and reproducibility, this method also has many disadvantages, including a relatively high production cost owing to the required vacuum conditions, low productivity due to the angular dependence of the deposition rate, and quite complicated fabrication process [12]. It is therefore still necessary to develop highly reflective DBRs with good thermal and mechanical stability by low-cost and simple processes.

Anodizing is an affordable and simple method used to fabricate porous anodic alumina (PAA) with a desired pore architecture that can be fully controlled by electrochemical parameters applied during the process [13,14]. A DBR-like structure is produced by pulse anodization, which involves the application of alternative low and high voltage (or current) pulses, which translate directly into low and high porosity layers. Porosity (*P*), in turn, determines the RI of the respective layers: RI decreases as *P* increases. Photonic materials are very sensitive to a slight change of the refractive index of a medium, which was used to fabricate a broad range of optical sensors [15,16,17,18]. The application of PAA-based PCs was limited to the visible (VIS) part of the spectrum due to the absence of corresponding crystals operating in mid-infrared (MIR) region. Yet, the MIR spectral range contains strong characteristic vibrational transitions of many important molecules and gases (e.g., CO_2_, CH_4_, CO, NO), which makes it crucial for applications in environmental monitoring, various industrial and security systems, or medical diagnosis. Despite the great potential of the MIR region, the optical components operating in this spectral domain are still under development. The situation is caused mainly by the lack of adequate and inexpensive optical materials, which would enable a precise manipulation of the infrared light. PAA-based PCs with photonic properties in UV-VIS were utilized in the detection of many biological molecules such as glucose, proteins, toxins, etc. [19,20,21]. Here, the biochemical interaction (e.g., binding) with the PC surface caused a change of the effective refractive index, which resulted in a shift of the PSB peak (the resonance wavelength shift per refractive index unit (nm/RIU)), which is proportional to the concentration of the bio-target. However, the utilization of PSBs’ shift as the sensing principle in MIR is impossible since many important organic and inorganic molecules have their absorbance bands there. The detection principle in MIR rely on the Beer-Lambert law [22,23,24]. A very important part of a typical sensor is a gas cell (an optical path, *l*) that guides light to interact with the gas [25]. An optical sensor operates by measuring the changes in the property of light as it passes through an analyte. To increase the sensor signal response long *l* is required (>3 cm [26]), which is in conflict with the emerging trend towards miniaturization of optical devises based on integration on-chip (lab-on-chip optical technology). The most promising strategy is based on the exploitation of enhancement layers, such as photonic crystals or optical cavities. Thanks to the slow light in the photonic structures, the required optical path length can be greatly reduced as slow light enhanced light–gas interaction and thus the absorption coefficient. Consequently, the sensor’s sensitivity can be significantly increased. Moreover, the approach enables the realization of miniature infrared gas sensors and provides potential applications for a real-time detecting trace of toxic gases in remote distance. Compared to complex and expensive techniques such as optical and electron-beam lithography methods, electrochemical synthesis offers a very attractive alternative to prepare cost-effective 1D PCs with repeatable optical properties.

Recently, we studied the influence of a broad range anodizing temperature (5–30 °C) on structural and photonic features of PAA-based DBRs [27]. It was revealed that above 10 °C the PSBs begin to drastically deteriorate. However, at 30 °C the material started to restore its photonic properties with distinct PSBs extending from the NIR to the MIR region. The preliminary results suggested the appearance of temperature induced reorganization of pore architecture into the DBR structure at 30 °C. In this work we analyze the influence of other anodizing parameters, such as magnitude of low (U_L_) and high (U_H_) voltage pulses, U_H_-U_L_ contrast, duration of the U_H_ and U_L_ pulses on the growth and optical characteristic of multilayered PAA during high-temperature-pulse anodization. The data demonstrates that the structural and photonic properties of PAA-based DBRs in the NIR-MIR spectral range can be substantially improved upon selection of the appropriate electrochemical parameters.

## 2. Materials and Methods

The PAA-based distributed Bragg reflector (DBR) structures were synthesized by a pulse anodization of aluminum. High-purity aluminum foil (99.9995% Al, Puratronic, Alfa-Aesar, Haverhill, MA, USA) with a thickness of about 0.25 mm was cut into specimens (2 cm × 1 cm). Before the anodization process, the Al foils were annealed under an argon atmosphere at 400 °C for 2 h. Then, the samples were degreased in acetone and ethanol and subsequently electropolished in a 1:4 mixture of 60% HClO_4_ and ethanol at 0 °C, under constant voltage of 25 V for 2.5 min. Next, the samples were rinsed with distilled water, ethanol, and dried. Al specimens were insulated at the back, and the edges were covered with acid resistant tape, which served as the anode. A Pt grid was used as a cathode and the distance between both electrodes was kept constant (ca. 5 cm). A large, 1 L electrochemical cell, a powerful low-constant-temperature bath (with temperature stability ±0.01 °C), and vigorous stirring (500 rpm) were employed in the anodizing process. Programmable DC power supply, model 62012P-600-8 Chroma, was used to control the applied voltage and the pulse parameters. The first anodization was carried out at 5 °C in 0.3 M C_2_H_2_O_4_ water-based solution, at 40 V, for 20 h. As obtained, alumina was chemically removed in a mixture of 6 wt% phosphoric acid and 1.8 wt% chromic acid at 60 °C for 3 h. Subsequently, pulse anodization with 20 cycles was conducted at the temperature range 26–29 °C. In general, a pulse sequence consisted of three steps: (1) a constant high voltage step (U_H_ = 50, 45, 40 V) (2) a gradual reduction of the voltage to low voltage at rates of 0.312, 0.234, 0.156, and 0.078 V/s; and (3) the anodization at a constant low voltage (U_L_ = 20, 15, 10 V). The duration of U_H_ = 50 V and U_L_ = 20 V voltages was varied: t_H_ = 360, 300, 240, 180 s, and t_L_ = 480, 420, 360, 300 s, respectively. After the pulse anodization was completed, the remaining aluminum substrate was selectively removed in a saturated solution of HCl/CuCl_2_.

Structural characterization of the PAA-based photonic structures was made using a field-emission scanning electron microscope FE-SEM (AMETEK, Inc., Mahwah, NJ, USA) equipped with energy dispersive X-ray spectrometer (EDS). The measurement of layer thickness was repeated three times at different points in the BSE (backscattered electrons) image of a given PAA sample and an average of the three measurements was taken to determine the initial and final d_H_ (the layers produced under U_H_ pulse) and d_L_ (the layers produced under U_L_ pulse) thickness. To obtain the geometrical parameters of the fabricated samples, Fast Fourier transforms (FFTs) were generated based on three SEM images taken at the same magnification for every sample and were further used in calculations with a freeware WSxM software (version 5.0). The average interpore distance (*D_c_*) was estimated as an inverse of the FFT’s radial average abscissa from three FE-SEM images for each sample. The average pore diameter (*D_p_*) was estimated from three FE-SEM images for each analyzed sample, using NIS-Elements software provided by Nikon Company, Tokyo, Japan.

The transmission spectra were measured with two instruments. The shortwave end of the spectrum (250–2500 nm) was measured using a Cary 5000 spectrometer with a DRA-2500 integrating sphere from Agilent Technologies Inc., Santa Clara, CA, USA. The longwave end of the spectrum (2500–25,000 nm) was measured using the Fourier-transform infrared (FTIR) spectrometer Alpha II from Bruker Corp., Billerica, MA, USA. The resolution of spectra was set to 1 nm in the shortwave range and 2 cm^−1^ in the longwave range.

## 3. Results and Discussion

**Figure 1:**
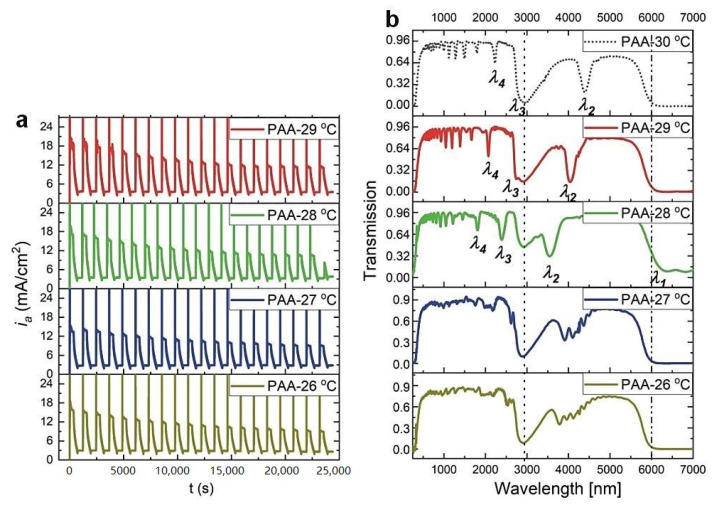
Current density (*i_a_*)—time (*t*) transients (**a**) and corresponding transmittance spectra (**b**) of PAA-based photonic structures anodized at the temperature range 29–26 °C with the following parameters: U_H_ = 50 V, t_H_ = 360 s, U_L_ = 20 V, t_L_ = 480 s, U_H_->U_L_ rate = 0.078 V/s, 20 cycles (the transmission spectrum for the PAA-30 °C sample is from ref. [27]).

Recently, it was demonstrated that the optical characteristics of PAA-based photonic material are extremely sensitive to anodizing temperature [27]. Particularly, the region between 25 and 30 °C appeared to be very interesting. It was observed that for the DBR prepared at 25 °C, photonic stopbands (PSBs) were split and hardly distinguishable, whereas the DBR synthesized at 30 °C demonstrated well-resolved and symmetric PSB peaks, shifted towards red part of the spectrum. Hence, the 25 to 30 °C region is further investigated in this work. In Figure 1, current density (*i_a_*)—time (*t*) transients along with the corresponding transmittance spectra of the multilayered PAA prepared during pulse anodization in the temperature range 26–29 °C, are shown (for comparison, the transmission spectrum of the sample anodized at 30 °C is also presented [27]).

As can be seen, up to 27 °C PSBs remain distorted. The peaks start to shape up again at 28 °C. Furthermore, the spectral positions of the bands shift towards the red part of the spectrum as the temperature increases. Although above 6 μm Al_2_O_3_ does not transmit light due to absorption by Al-O bond vibrations [28,29], in the sample fabricated at 28 °C, just above the edge of the transmission, a vague first order PSB (*λ*_1_) can be also discernable. The resonance peaks were assigned to different orders of a given stop band based on the Bragg-Snell equation [30], assuming the angle of incidence *θ* ~ 0 (*λ*_i_, i = 1–4, correspond to 1–4 orders of PSB). The broad peak at around 3000 nm, present in all spectra and marked by vertical, black, dotted line, originate from the OH group vibrations of adsorbed water [31].

**Figure 2:**
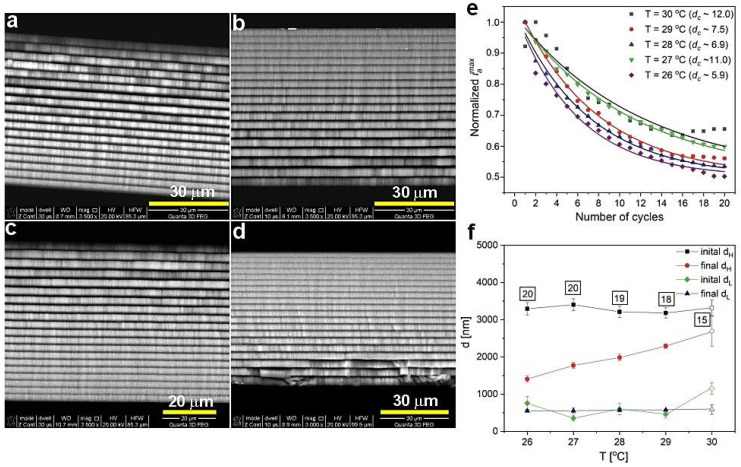
BSE images of PAA-29 °C (**a**), PAA-28 °C (**b**), PAA-27 °C (**c**), PAA-26 °C (**d**) samples; normalized *i_a_^max^* as a function of number of cycles (**e**); initial and final thickness of d_H_ and d_L_ layers as a function of anodizing temperature (**f**); the number in black squares indicate the number of d_H_+d_L_ pairs in the respective samples (the normalized *i_a_^max^* and layer thickness for the PAA-30 °C sample [27] are also shown for comparison).

In Figure 2, BSE images of the PAA multilayered structure prepared at 29 (a), 28 (b), 27 (c), and 26 °C (d) are demonstrated, where the material density contrast between alternate and subsequent d_H_ (formed under U_H_) and d_L_ (formed under U_L_) layers are clearly visible. As can be seen in Figure 1a, the current recovery peak (the *i_a_^max^* was determined as in the work [27]) after application of subsequent U_H_ pulses decreased with the number of cycles, which indicates a decrease of the total amount of charge involved in the anodization reaction. The decreasing amount of the net amount of charge, in turn, means that the thickness of the d_H_ segments grown at the following U_H_ pulses will be gradually reduced. In Figure 2e, the normalized *i_a_^max^* as a function of number of cycles is presented for the samples synthesized at different temperature. The *i_a_^max^* course can be well described by a simple first order exponential decay (*y* = *A*_1_ exp(−*x*/*d_c_*) + *y*_0_, where *A*_1_ is the amplitude, *y*_0_ is the offset term that represents residual *i_max_*, and *d_c_* is the decay constant). In this particular case, the *d_c_* indicates the U_H_ pulse at which the course reaches 1/e or ≈37% of the difference between the initial (*y*_0_) and final (*y*) steady state values. *y*_0_ value was fixed to 0.5 for all samples, to assure the same fitting range from the maximum value up to the point where the *i_a_^max^* reaches the half of its maximum value. The larger the *d_c_* value, the more efficient is the current recovery under subsequent U_H_ pulses, and thus a better selection of the electrochemical parameters to maintain a stable pulse anodization is achieved. Based on this analysis, the processes conducted at 27 (*d_c_* = 11) and 30 °C (*d_c_* = 12) were better designed than the other ones (Figure 2e). This is, however, not compatible with optical analyses (Figure 1b), where the sample produced at 27 °C did not show features characteristic of the DBR structure. It thus seems that the variability of the *d_c_* parameter within the range 6–12 does not significantly affect the optical characteristics of the multilayer PAA structure. The only parameter that matters in this case is the temperature: the multilayered PAA transforms into the DBR only at relatively high temperature (28–30 °C). The statement is also supported by the difference between initial (mean thickness of the first three layers) and final (mean thickness of the last three layers) d_H_. Figure 2d shows that this difference weakens as the anodizing temperature increases. However, it has to be noticed that starting from 28 °C, the number of d_H_+d_L_ pairs decreases (the numbers are shown in square frames in Figure 2f). This means that the thickness of initial d_H_ was not determined for exactly the first three (1–3) layers, but for 2–4, or even 6–8 ones, which might have decreased slightly its average value. Consequently, the difference between initial and final d_H_ thickness is a bit underestimated. The lack of several layers may be due to non-equilibrated rate- and diffusion-controlled processes at a high anodization temperature [27]. However, it is also very probable that the first layers are simply falling off from the stack after the synthesis. The BSE image of the sample PAA-26 °C (Figure 2d), for instance, suggests that the first segments are more brittle and vulnerable to exfoliation. The exfoliation may occur during the Al dissolution in the mixture of HCl acid and CuCl_2_ or during the preparation of the samples to analysis (the samples have to be broken in order to analyze their cross-sectional). While the thickness of the d_H_ layers increased substantially with the anodizing temperature, the d_L_ for the samples anodized at 26–29 °C apparently stayed on more or less the same level (Figure 2f).

**Figure 3:**
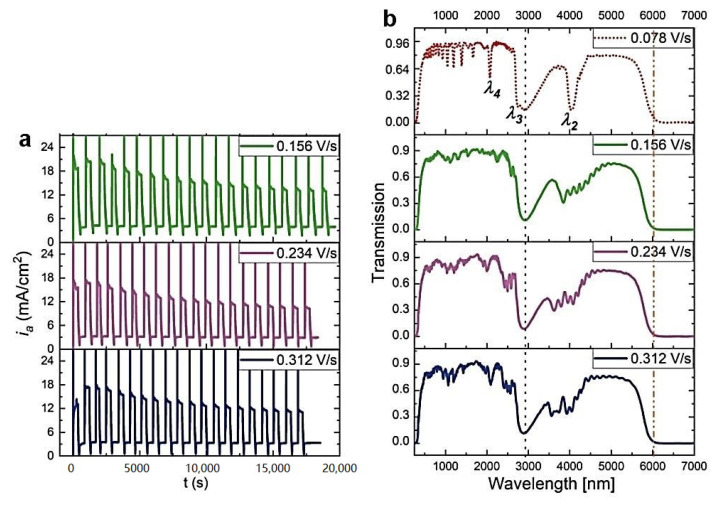
The *i_a_*(*t*) curves recorded during pulse anodization at 29 °C (other parameters: U_H_ = 50 V, t_H_ = 360 s, U_L_ = 20 V, t_L_ = 480 s, 20 cycles) with increasing U_H_->U_L_ rate from 0.156 V/s down to 0.312 V/s (**a**), the corresponding transmission spectra (**b**). The transmission spectra of the PAA-29 °C sample synthesized with the U_H_->U_L_ rate of 0.078 V/s is also shown for comparison.

**Figure 4:**
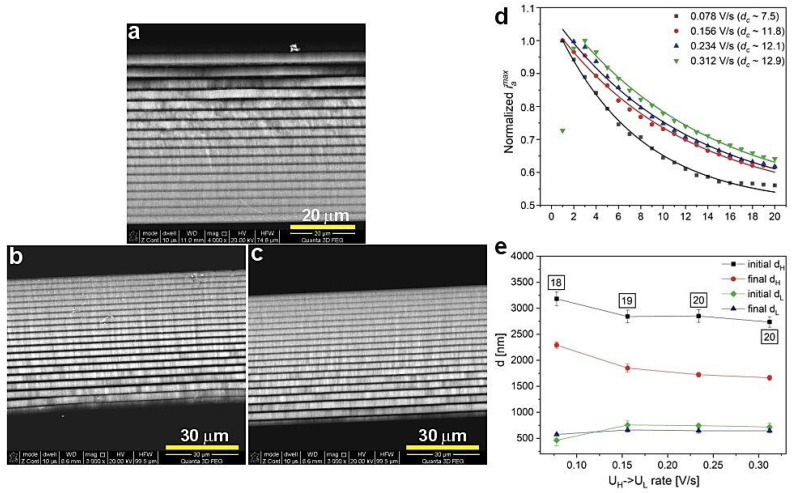
BSE images of the PAA synthesized at 29 °C with different U_H_->U_L_ rate: 0.156 (**a**), 0.234 (**b**), and 0.312 V/s (**c**), normalized *i_a_^max^* as a function of number of cycles (**d**), and initial and final d_H_ and d_L_ layer thickness as a function of U_H_->U_L_ rate (**e**); in black squares—the number of d_H_+d_L_ pairs.

Previously, an improvement of the geometrical and photonic properties of the PAA-based DBR produced at 30 °C upon increasing the U_H_->U_L_ rate was observed [27]. The same protocol was applied to the PAA sample anodized at 29 °C. As visible in Figure 3, the increase of this parameter resulted in a complete distortion of the PSBs. Already under application of the U_H_->U_L_ = 0.156 V/s, the transmission spectrum lacks the features typical for the DBR structure. This is quite surprising considering the small (only by 1 °C) temperature drop. Figure 4a–c shows typical multilayer structures with an increased number of d_H_+d_L_ layers as the U_H_->U_L_ rate increases. The *d_c_* determined for the samples prepared with the increased U_H_->U_L_ rates is even larger than the *d_c_* determined for the sample anodized with the lowest U_H_->U_L_ rate = 0.078 V/s (Figure 4d). Again, this might suggest that the *d_c_* within the 8–13 range does not influence the optical performance of the PAA material. As previously observed [27], the thickness of the initial and final d_H_ layers decreases as the U_H_->U_L_ increases (Figure 4e), whereas d_L_ demonstrates a quite stable behavior. Taking into account the possible exfoliation of the first two layers (in the sample anodized under the 0.078 V/s rate) and the first one (in the sample anodized under the 0.156 V/s rate) from the potential 20-layer stack, the difference between the initial and final d_H_ seems to remain stable. What is, therefore, the reason causing the deterioration of the PSBs in these samples? It is possible that the temperature of 29 °C is already too low to maintain good pore reorganization when the change from U_H_ to U_L_ is too fast.

**Figure 5:**
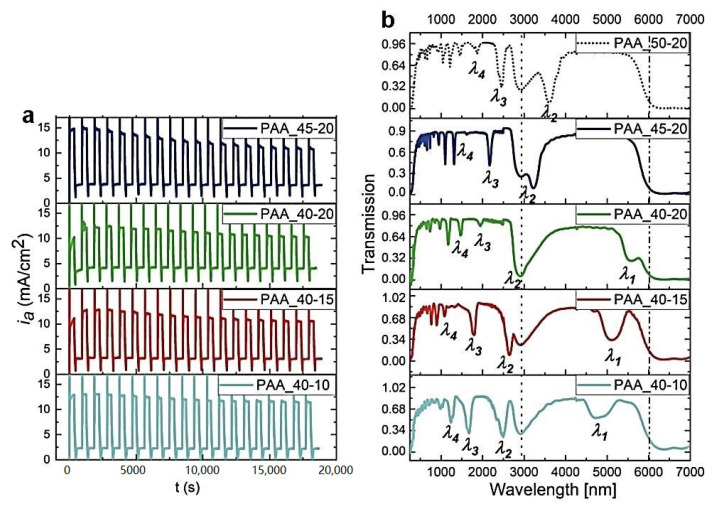
The *i_a_*(*t*) curves recorded during pulse anodization at 30 °C under different U_H_ and U_L_ pulses [V] (PAA_U_H_-U_L_, other applied parameters: t_H_ = 360 s, t_L_ = 480 s, U_H_->U_L_ drop rate = 0.234 V/s, 20 cycles) (**a**) and the corresponding transmission spectra (the transmission spectra for the PAA_50-20 is taken from ref. [27] for comparison) (**b**).

Since lowering the temperature already by 1 °C has such detrimental effect on the optical characteristics of PAA multilayered structures, we decided to analyze the influence of other anodizing parameters on the PAAs prepared at 30 °C using an optimal U_H_->U_L_ rate (0.234 V/s), as determined based on the previous investigations [27]. At first, the effect of U_H_ and U_L_ values and U_H_-U_L_ contrast of the geometrical and optical properties of multilayer PAAs was analyzed. In Figure 5, the *i_a_*(*t*) curves along with the corresponding transmission spectra recorded for PAA anodized under different U_H_ and U_L_ pulses, are shown. As can be seen, the photonic characteristics typical for the DBR structure are maintained for all the studied samples. Upon a decrease of U_H_ from 50 to 40 V and simultaneously a decrease of the U_H_-U_L_ contrast, the PSB peaks shift towards the blue part of the spectrum, while keeping their intensity and symmetry. Additionally, in the spectrum of the PAA_40-20 sample, a clear *λ*_1_ band is discernable at ~5560 nm. Decreasing U_L_ from 20 to 10 V, and at the same time increasing the U_H_-U_L_ contrast, causes a further shift of the PSB peaks towards shorter wavelengths. In general, the PAA-based DBRs in this batch of the samples preserve their good optical characteristics, which is particularly visible when monitoring the *λ*_2_ mode behavior. It can be stated that the best optical performance demonstrates the PAA_40-15 sample for which both *λ*_1_ and *λ*_2_ bands are symmetric and intensive (T = 0.3 and 0.07, respectively). In the PAA_40-10 sample, the *λ*_1_ and *λ*_2_ peaks appear to weaken and become less symmetric. The intensity of higher photonic modes (e.g., *λ*_3_ and *λ*_4_) varies from sample to sample. It is well known that higher interference modes are more sensitive to possible structural imperfections or boundary conditions [32]. Therefore, any deviation from ideal multilayered structure (e.g., irregular layer thickness) or some damages in the structure, is more noticeable in higher modes.

**Figure 6:**
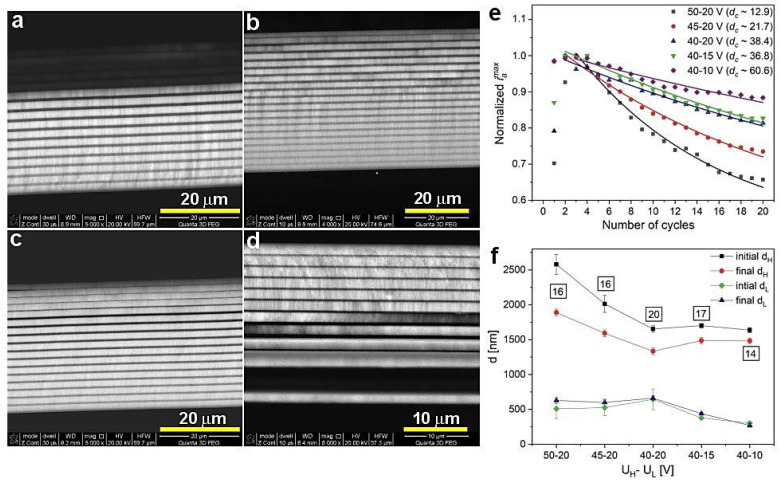
BSE images of the samples PAA_45-20 (**a**), PAA_40-20 (**b**), PAA_40-15 (**c**), PAA_40-10 (**d**), normalized *i_a_^max^* (the data for the PAA_50-20 sample comes from ref. [27]) as a function of number of cycles (**e**) and initial and final d_H_ and d_L_ layer thickness for the presented samples (**f**); in black squares—the number of d_H_+d_L_ pairs.

Figure 6 demonstrates BSE images of the PAA_45-20 (a), PAA_40-20 (b), PAA_40-15 (c), and PAA_40-10 (d) samples, normalized *i_a_^max^* as a function of number of cycles (e), and initial and final d_H_ and d_L_ layer thickness (f) of the respective samples. For this group of the samples there is an obvious trend in the *d_c_* behavior: the *d_c_* gradually increases as U_H_ and then U_L_ decreases (Figure 6e). This trend is followed by the decrease of the difference between initial and final d_H_ values (Figure 6f). In the PAA_50-20, PAA_45-20, and PAA-40-20 samples, the different decreases are owed to the U_H_-U_L_ contrast decrease and is the smallest for the PAA_40-20 sample. This sample is also built out of complete 20 d_H_+d_L_ segments, suggesting that the actual difference between initial and final d_H_ layers in the samples PAA_50-20 and PAA_45-20 (16 d_H_+d_L_ pairs) is even greater than shown in Figure 6f. The difference seems to decrease as well for the subsequent PAA-40-15 and PAA-40-10 samples, despite the increase of the U_H_-U_L_ contrast. This is, however, an apparent effect coming from the reduced number of the d_H_+d_L_ pairs. The sample PAA_40-10 consists of only 13 d_H_+d_L_ segments, so initial d_H_ value was actually determined for the 8th to 10th layer, not for the first three (1–3) real layers. The BSE image of the PAA_40-10 sample (Figure 6d) strongly indicates that this DBR structure is more susceptible to exfoliation as compared to other samples, which is most likely caused by its higher brittleness. The higher fragility, in turn, may result from relatively low values of potential pulses applied during anodization (U_L_ is only 10 V). Owing to the large structural imperfection, the PAA_40-10 DBR demonstrates also a slightly worse optical characteristics (Figure 5b), as compared to other samples from this batch. Contrary to the d_H_ behavior, both initial and final d_L_ layers are stable in the PAA_50-20, PAA_45-20, and PAA-40-20 samples, and go down in the PAA-40-15 and PAA-40-10 samples due to the U_L_ decrease.

**Figure 7:**
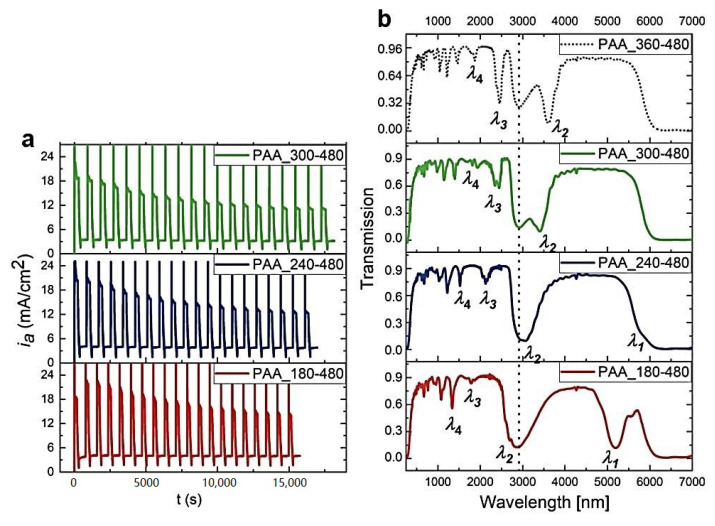
The *i_a_*(*t*) curves recorded during pulse anodization at 30 °C, under U_H_ of different t_H_ [s] (PAA_t_H_-t_L_, other parameters: U_H_ = 50 V, U_L_ = 20 V, U_H_->U_L_ rate = 0.234 V/s, 20 cycles) (**a**) and the corresponding transmission spectra (the transmission spectra for the PAA_360-480 is taken from ref. [27] for comparison) (**b**).

Next, the influence of the U_H_ and U_L_ pulse duration on the multilayer PAA formation and its optical performance was tested. In Figure 7, the *i_a_*(*t*) curves and corresponding transmission spectra of the PAA-based DBRs prepared under U_H_ with decreasing duration (t_H_) are shown. Owing to the shorter t_H_, the d_H_ segments become progressively thinner. This results in a blue shift of PSBs. When U_H_ pulses with t_H_ = 240 s are applied, a weak outline of the *λ*_1_ peak is already visible in the transmission spectrum. The *λ*_1_ band becomes well-resolved in the PAA-based DBR anodized with t_H_ = 180 s.

**Figure 8:**
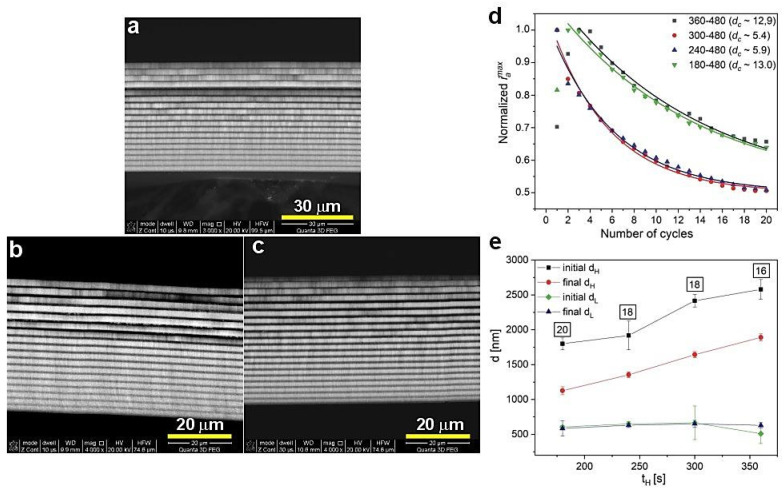
BSE images of the samples PAA_300-480 (**a**), PAA_240-480 (**b**), PAA_180-480 (**c**), normalized *i_a_^max^* as a function of number of cycles (**d**) and initial and final layer d_H_ and d_L_ thickness as a function t_H_ (**e**); in black squares—the number of d_H_+d_L_ pairs.

The BSE images of the PAA_300-480, PAA_240-480, and PAA_180-480 are shown in Figure 8a, b, and c, respectively. The course of *i_a_^max^* is comparable for all samples from this batch (Figure 8d). The *d_c_* parameter is within the range of 5 to 13. The decrease of d_H_ segments upon shortening of the U_H_ duration is well presented in Figure 8d. Moreover, with shorter t_H,_ the number of d_H_+d_L_ layers increases; the PAA_180-480 DBR is built out of complete 20 d_H_+d_L_ segments. Taking into account the lower number of the d_H_+d_L_ pairs in other DBRs (Figure 8e) it can be concluded that the difference between initial and final d_H_ remains pretty stable. Therefore, the analysis presented in Figure 8 confirms similar optical characteristics for all these samples: the PSBs are well-resolved (although some *λ*_2_ bands are overlapped with water peak), symmetric, and intensive (Figure 7).

**Figure 9:**
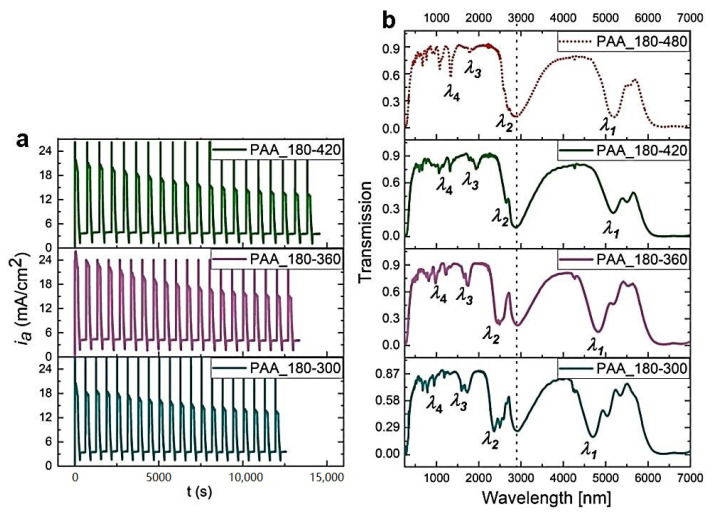
The *i_a_*(*t*) curves recorded during pulse anodization at 30 °C, under U_L_ of different t_L_ [s] (PAA_t_H_-t_L_, other parameters: U_H_ = 50 V, U_L_ = 20 V, U_H_->U_L_ drop rate = 0.234 V/s, 20 cycles) (**a**) and the corresponding transmission spectra (the transmission spectra for the PAA_180-480 is shown for comparison) (**b**).

**Figure 10:**
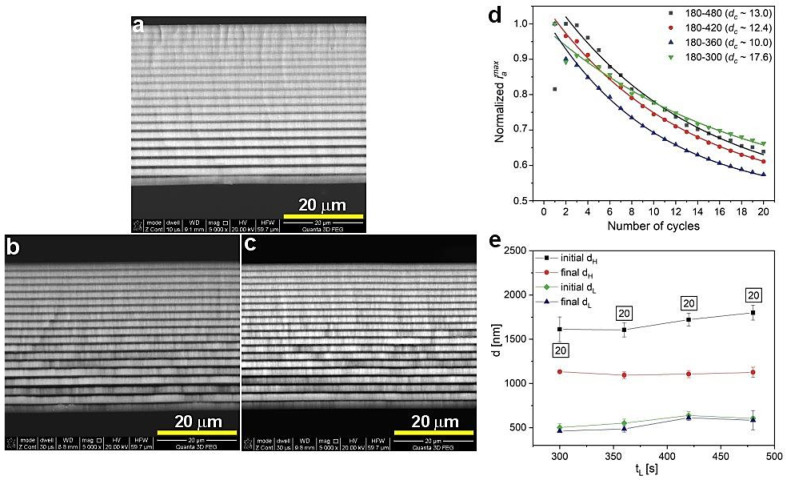
BSE images of the samples PAA_180-420 (**a**), PAA_180-360 (**b**), PAA_180-300 (**c**), normalized *i_a_^max^* as a function of number of cycles (**d**) and initial and final layer d_H_ and d_L_ thickness as a function t_L_ (**e**); in black squares—the number of d_H_+d_L_ pairs.

In Figure 9, the *i_a_*(*t*) curves and corresponding transmission spectra of the PAA-based DBRs fabricated under U_L_ with decreasing duration (t_L_) are demonstrated. With decreasing t_L_, a small blue shift of PSBs is observed. However, these DBRs seem to start losing their good photonic properties: the PSBs are becoming progressively broadened and start to severely split as t_L_ decreases (Figure 9b). The samples PAA_180-480, PAA_180-420, PAA_180-360, and PAA_180-300 consist of full 20 d_H_+d_L_ pairs (Figure 10a–c) and the *d_c_* parameter varies within the range 10–18 (Figure 10d). Moreover, the difference between initial and final d_H_ appears to be stable for all samples in this series or even slightly decreases when shortening the t_L_ (Figure 10e). The latter observation may indicate more efficient diffusional processes occurring when t_L_ is shortened. Hence, both current behavior and determined geometrical features do not say much about the source of the slight but visible worsening of optical performance. However, the changes are evident upon shortening t_L_. It can thus be supposed that the resulted thinner d_L_ segments (Figure 10e) somehow affect an overall multilayer structure, which becomes to a certain degree distorted. The distortion can for instance be linked with worse pore arrangement and circularity in the d_L_ layers formed under shorter U_L_ pulses. It was demonstrated that these parameters depend on the anodizing time: the longer the anodization time, the better the pore arrangement and circularity [33].

Summarizing, the PAA-based DBR is extremely sensitive to pulse anodization temperature. In the temperature >10 °C and up to 27 °C the typical DBR features are not observed in transmission spectra. Above 27 °C, the PAA multilayer structure transforms into the DBR photonic crystal, which is manifested by the appearance of distinct, well-resolved, and symmetric peaks in the transmission spectra. Within the conditions applied in this study, optimal PAA-based DBRs are produced at 30 °C using a U_H_->U_L_ drop rate close to 0.234 V/s. The optical properties of the DBRs formed under the high-temperature pulse-anodization can be tuned by changing other anodization parameters, such as U_H_, U_L_, U_H_-U_L_ contrast, and t_H_. Decreasing t_L_ causes progressive deterioration of the optical characteristics of the photonic crystals. On the other hand, too low U_L_ voltage contributes to the formation of too much brittleness of the multilayer PAA material and, consequently, harsh exfoliation of initial d_H_ and d_L_ layers.

DBR performance (i.e., transmittance and PSB bandwidth) is determined by the number of pairs as well as the refractive index contrast between low and high RI layers [34]. A larger contrast and higher number of pairs can lead to lower transmittance and wider PSBs. In the electrochemical production of PAA-based DBR at relatively high temperature, the most challenging task is to control a uniform growth of d_H_+d_L_ pairs, which is directly proportional to the net amount of the charge involved in the anodization reaction. The non-uniform layer formation can be partially counteracted by decreasing U_H_ and U_L_ values and U_H_-U_L_ contrast. Nevertheless, it is still difficult to maintain a steady current recovery (constant value of *i_a_^max^*) during the whole process. Therefore, to master the production of PAA-based photonic crystals with the desired photonic properties in NIR-MIR spectral range, this experimental approach will be modified in future studies. The PAA-based DBRs with the best optical properties observed in this work are presented in Table 1 with the anodization parameters used to produce those samples.

**Figure 11:**
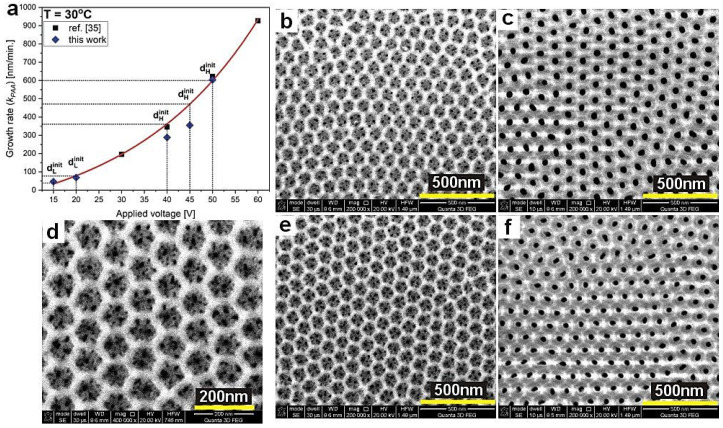
PAA growth rate (*k_PAA_*) as a function of applied voltage for T = 30 °C (**a**), SEM images of the d_L_^init^ and d_H_^init^ layers (top views) formed under U_L_ = 20 V (**b**), U_H_ = 50 V (**c**), U_L_ = 15 V (**d**,**e**), U_H_ = 40 V (**f**) pulses at 30 °C.

The observed optical resonances generated by the samples gathered in the Table 1 were also verified theoretically assuming a DBR structure built of uniform segments that are formed under equilibrated conditions (no rate and diffusion limitations). These conditions are operative during application of the first U_H_ and U_L_ pulses in a given pulse sequence. Accordingly, the thickness of the initial d_H_ and d_L_ layers can be considered as the one that would be repeated in every cycle if no rate- and/or diffusion-limited processes occurred, thereby assuring the same amount of charge flowing in each cycle. However, owing to the lack of the first one to three layers in some of the multilayer stacks, the thickness of the initial d_H_ and d_L_ segments (d_H_^init^ and d_L_^init^, respectively) was determined with support of the data established in ref. [35]. In Figure 11a, the growth rate (*k_PAA_*) of PAA layers formed under pulse anodization (this work) is compared with that determined in [35]. It can be seen that owing to the lack of the first layers in the DBR stacks, the *k_PAA_* of d_H_^init^ segments formed at 40 and 45 V were underestimated. However, the *k_PAA_* of the d_L_^init^ segments formed at 15 and 20 V and that of d_H_^init^ formed at 50 V (the PAA_180-480 sample with the full 20 d_H_+d_L_ pairs) agreeed very well with the trend established in ref. [35]. The d_H_^init^ and d_L_^init^ thicknesses for the DBR produced at 29 °C was measured directly on the sample PAA_29 °C. In order to determine refractive indices of the d_H_^init^ and d_L_^init^ layers, porosity of PAA layers was calculated using the following equation [36]:

(1) $$P=\frac{\pi }{2\sqrt{3}}{\left(\frac{D_{p}}{D_{C}}\right)}^{2}=0.907{\left(\frac{D_{p}}{D_{C}}\right)}^{2},$$

where *D_p_* is pore diameter and *D_c_* is the interpore distance. To measure *D_p_* and *D_c_* of d_H_ layers, two-period stacks were synthesized using the following pulse sequence: U_H_-U_L_-U_H_-U_L_, whereas the U_L_-U_H_-U_L_-U_H_ inverse pulse sequence was used to analyze the d_L_ layers. In Figure 11b–f, SEM images of d_H_ and d_L_ layers (top views) of the selected samples are shown. It can be seen that the d_L_ layers have a complex structure where a few smaller pores are located within a larger hexagonal cell (about 10 pores per cell; the image showing larger magnification of the d_L_ layer in Figure 11d). The hexagonal cells form an almost perfect honeycomb structure. Since it was not possible to determine both pore diameter and interpore distance from such complex images using the protocol described in the Section 2, the *D_d_^L^* and *D_c_^L^* were derived from the formulas developed in the work [37]. All geometrical parameters of PAA layers are gathered in Table 2.

Having porosity values, the refractive indices of the d_H_^init^ and d_L_^init^ segments (*n_H_* and *n_L_*, respectively) were determined based on the Bruggeman’s effective medium theory [38], and the effective refractive index (*n_eff_*) of each double layer was calculated using the following formula [39]:

(2) $$n_{eff}=\frac{n_{H}d_{H}^{\mathrm{init}}+n_{L}d_{L}^{\mathrm{init}}}{d_{H}^{\mathrm{init}}+d_{L}^{\mathrm{init}}}.$$

Bragg-Snell law gives the spectral positions of photonic stop bands (PSBs) [30]:

(3) $$m\lambda_{m}=2d\sqrt{n_{eff}^{2}-n_{air}^{2}{sin}^{2}\theta },$$

where *λ* is the wavelength of a photonic stop band (PSB), *m* is the order of the PSB, *d* is the periodicity (*d* = d_H_^init^ + d_L_^init^), *θ* is the angle of incidence (*θ* ~ 0 in the studied cases), *n_eff_* is the effective refractive index, and *n_air_* is the refractive index of air. The optical constants and the calculated PSBs are presented in Table 3.

As can be seen, the resulting spectral positions of *λ*_1_ and *λ*_2_ are shifted towards longer wavelengths as compared to the observed ones (*λ*_1_^obs^ and *λ*_2_^obs^), suggesting that the real *n_eff_* and/or *d* values of the studied DBRs are smaller. Therefore, the PSBs spectral positions were also determined, taking into account the mean values of both initial and final d_H_ and d_L_ layers ($\bar{d_{H}}$ and $\bar{d_{L}}$, respectively, Table 2; *d* = $\bar{d_{H}}$ + $\bar{d_{L}}$ in this case). The corresponding PSBs (*λ*′_1_ and *λ*′_2_) are collected in Table 3. The *n_eff_* remained practically stable when d_H_^init^ and d_L_^init^ were substituted by the $\bar{d_{H}}$ and $\bar{d_{L}}$ in Equation (2): its values changed only in the third decimal place. Now, the *λ*′_1_ and *λ*′_2_ are much closer the observed ones (*λ*_1_^obs^ and *λ*_2_^obs^). The match would be even better if an average porosity of all 20 d_H_ and d_L_ pairs was known. The porosity of subsequent segments may also vary: in the 20-period DBR stacks the initial segments most likely have larger pore diameters—and thus porosity—than the final ones due to the prolonged stay in the oxalic solution. Consequently, the average *P_H_* and *P_L_* would be larger than the ones determined for the two-period stacks. The larger porosity, in turn, contributes to smaller refractive indices of porous layers and thus to smaller *n_eff_*. In Table S1 (Supplementary Materials), simulated optical spectra of the PAA-29 °C, PAA_45-20, PAA_40-15, and PAA_180-480 DBRs are demonstrated for d_H_^init^, d_L_^init^, and the *n_H_* and *n_L_* determined based on the layer porosities. The optical spectra are compared with those simulated for the $\bar{d_{H}}$ and $\bar{d_{L}}$ and for *n_H_* and *n_L_* chosen to make PSBs match with the the *λ*_1_^obs^ and *λ*_2_^obs^. Based on this analysis it appears that the actual *n_H_* and *n_L_* of the DBRs are in the ranges 1.28–1.41 and 1.18–1.24, respectively. The effective refractive index, in turn, lies within the range 1.24–1.39.

## 4. Conclusions

In this work, the influence of various electrochemical parameters on the production of PAA-based DBRs during high-temperature-pulse anodization was studied. It was observed that the process and resulting DBR properties are very sensitive to anodizing temperature: lowering the temperature from 30 to 27 °C brings about drastic changes in optical performance of the DBRs. The multilayered PAA fabricated at 27 °C did not demonstrate optical properties typical for DBR. Upon decreasing U_H_ and U_L_ potential and U_H_-U_L_ contrast, the current recovery (*i_a_^max^*) after application of subsequent U_H_ pulses started to stabilize, which was also manifested by a smaller difference between initial and final d_H_ thickness and a better DBR performance. The optimal U_H_-U_L_ contrast at 30 °C is 25 V: in the PAA_40-15 sample both *λ*_1_ and *λ*_2_ bands in the transmission spectra are well-resolved, intensive and symmetric. Furthermore, shortening the U_H_ pulse duration results in a progressive shift of PSBs towards blue part of the spectrum without signs of PSB deterioration. The sample PAA_180-480 generates well-developed peaks in the MIR (*λ*_1_ = 5183 nm) and NIR (*λ*_2_ = 2697 nm) region. The effective refractive indices *n_eff_* for the best performing samples lie within the range 1.24–1.39 and are smaller than the ones calculated for perfect multilayer structures of uniform thickness and porosity. Despite the obvious improvement of DBR properties in the NIR-MIR region by modulation of electrochemical parameters, there is still a problem with a full control over the homogeneous formation of d_H_+d_L_ pairs. Since the amount of charge determines the thicknesses of anodized segments at given anodization potentials, the problem with the non-uniform growth of d_H_+d_L_ pairs can be solved, for instance, by designing potential pulse sequences with a given amount of charge for U_H_ and U_L_ pulses. In this approach, the duration of subsequent U_H_ and U_L_ pulses would be adjusted to the time needed to reach the same amount of charge for each U_H_ and U_L_ pulses—this work is now in progress.

## Figures and Tables

**Table 1:** Electrochemical parameters applied during a 20-cycle pulse anodization at relatively high temperatures, which was used to synthesize PAA-based DBRs with the best optical properties along with the observed PSBs (T stands for transmission intensity).

Sample	Temperature [°C]	U_H_ [V]	t_H_ [s]	U_L_ [V]	t_L_ [s]	U_H_->U_L_ [V/s]	*λ*_1_^obs^ [nm] (T)	*λ*_2_^obs^ [nm] (T)
PAA-29 °C	29	50	360	20	480	0.078	-	4042 (0.14)
PAA_45-20	30	45	360	20	480	0.234	-	3220 (0.08)
PAA_40-15	30	40	360	15	480	0.234	5110 (0.31)	2645 (0.08)
PAA_180-480	30	50	180	20	480	0.234	5183 (0.10)	2697 (0.20)

**Table 2:** Geometrical parameters of PAA layers: *D_d_^H^* and *D_d_^L^*—pore diameters, *D_c_^H^* and *D_c_^L^*—interpore distances, *P_H_* and *P_L_*—porosity of the d_H_ and d_L_ segments, respectively; d_H_^init^ and d_L_^init^ are the initial segments formed under first U_H_ and U_L_ pulses, respectively; $\bar{d_{H}}$ and $\bar{d_{L}}$ are the mean values of both initial and final thicknesses of d_H_ and d_L_ layers, respectively

Sample	*D_d_^H^* [nm]	*D_d_^L^* [nm]	*D_c_^H^* [nm]	*D_c_^L^* [nm]	*P_H_* [%]	*P_L_* [%]	d_H_^init^ [nm]	d_L_^init^ [nm]	$\bar{d_{H}}$ [nm]	$\bar{d_{L}}$ [nm]
PAA-29 °C	36 ± 3	25 ± 5	105 ± 8	48 ± 5	11 ± 3	24 ± 12	3182	574	2737	518
PAA_45-20	38 ± 5	25 ± 5	103 ± 7	48 ± 5	13 ± 5	24 ± 12	2826	618	1803	562
PAA_40-15	39 ± 7	21 ± 5	98 ± 7	42 ± 5	14 ± 5	21 ± 13	2165	310	1594	308
PAA_180-480	43 ± 4	25 ± 5	105 ± 5	48 ± 5	15 ± 4	24 ± 12	1798	618	1463	595

**Table 3:** Optical indices (*n_H_* and *n_L_* are refractive indices of the respective d_H_^init^ and d_L_^init^ segments, *n_eff_* is the effective refractive index) and PSBs calculated for d_H_^init^ and d_L_^init^ (*λ*_1_ and *λ*_2_) and for $\bar{d_{H}}$ and $\bar{d_{L}}$ (*λ*′_1_ and *λ*′_2_).

Sample	*n_H_* *	*n_L_* *	*n_eff_*	*λ*_1_ [nm]	*λ*_2_ [nm]	*λ*′_1_ [nm]	*λ*′_2_ [nm]
PAA-29 °C	1.67	1.55	1.65	12,408	6204	10,748	5374
PAA_45-20	1.65	1.55	1.63	11,242	5621	7692	3846
PAA_40-15	1.64	1.57	1.63	8076	4038	6197	3099
PAA_180-480	1.63	1.55	1.61	7779	3890	6612	3306

* determined based on Bruggeman’s effective medium theory [38].

## Supplementary Materials

The following are available online at https://www.mdpi.com/1996-1944/13/24/5622/s1, Table S1: Simulated optical spectra of the PAA-29 °C, PAA_45-20, PAA_40-15, and PAA_180-480 DBRs.
