# Supplementary material for: Fabrication of Porous Anodic Alumina (PAA) by High-Temperature Pulse-Anodization: Tuning the Optical Characteristics of PAA-Based DBR in the NIR-MIR Region
Source: Materials (Basel). 2020 Dec 9;13(24):5622. doi: 10.3390/ma13245622 (PMC7764316; doi:10.3390/ma13245622)
Supplement: Supplementary file 1 [file materials-13-05622-s001.zip › materials-1017985-supplementary.pdf]

materials

MDPI

Supplementary Materials

Fabrication of Porous Anodic Alumina (PAA) by High-Temperature Pulse-Anodization: Tuning the Optical Characteristics of PAA-based DBR in the NIR-MIR Region

Ewelina Białek [1], Maksymilian Włodarski [2] and Małgorzata Norek [1,*]

[1] Institute of Materials Science and Engineering, Faculty of Advanced Technologies and Chemistry, Military University of Technology, Str. gen Sylwestra Kaliskiego 2, 00-908 Warsaw, Poland; ewelina.bialek2@gmail.com

[2] Institute of Optoelectronics, Military University of Technology, Str. gen. Sylwestra Kaliskiego 2, 00-908 Warsaw, Poland; maksymilian.wlodarski@wat.edu.pl

* Correspondence: malgorzata.norek@wat.edu.pl;

Received: date; Accepted: date; Published: 9 December 2020

Table S1 Simulated optical spectra of the PAA-29 °C, PAA_45-20, PAA_40-15, and PAA_180-480 DBRs performed using WOLFRAM Demonstration Project: https://demonstrations.wolfram.com/MultilayerPhotonicBandgap/ (in this simulation: $t_\alpha = 2d_H$ and $t_\beta = 2d_L$). Two cases were considered:

1. taking into account d_H^{init} and d_L^{init} and n_H and n_L values determined based on PAA characterization,

2. taking into account $\overline{d_H}$ and $\overline{d_L}$ and assumed n_H and n_L values to make PSBs match with the observed resonances λ_1^{obs} and λ_2^{obs}.

	**1.**	**2.**
**PAA-29 °C**	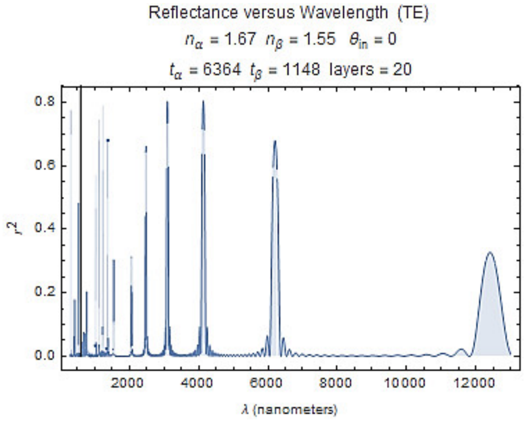	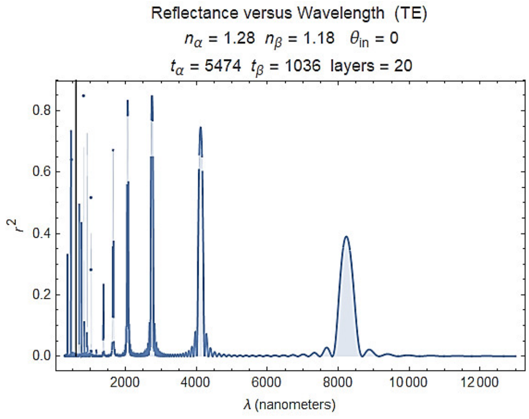

<table>
<tr>
<td>PAA_45-20</td>
<td>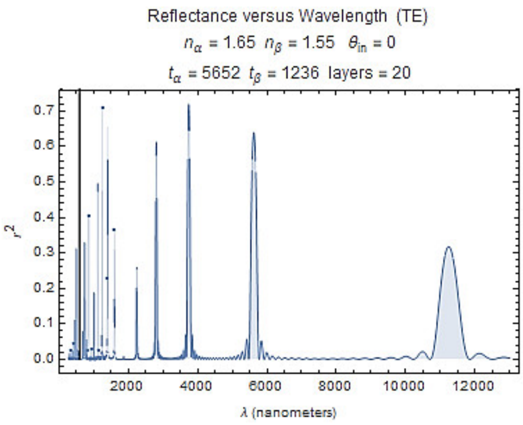
</td>
<td>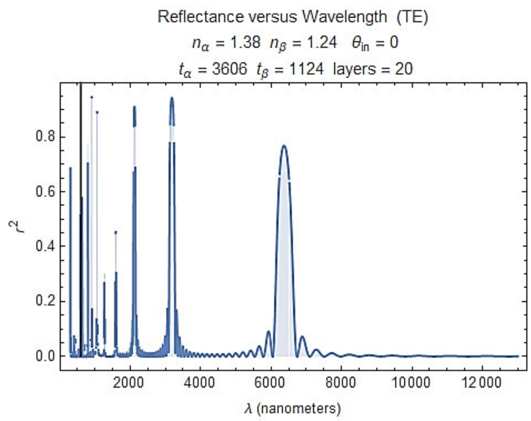
</td>
</tr>
<tr>
<td>PAA_40-15</td>
<td>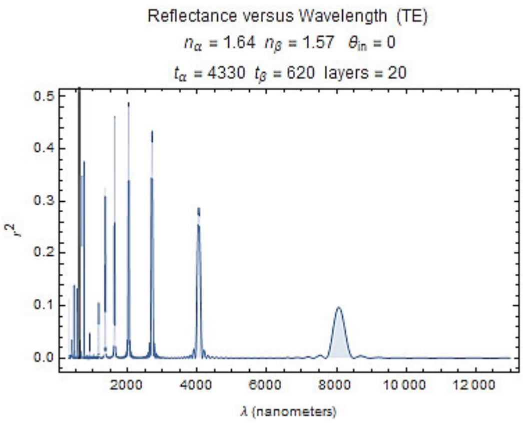
</td>
<td>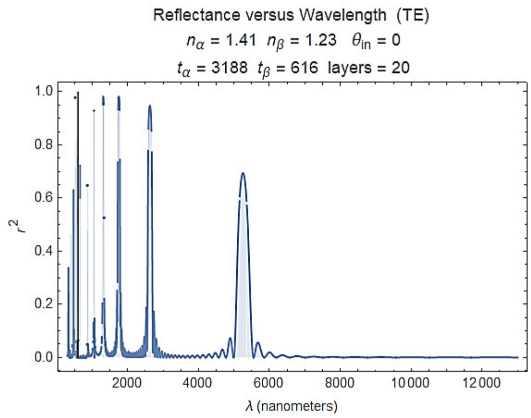
</td>
</tr>
<tr>
<td>PAA_180-480</td>
<td>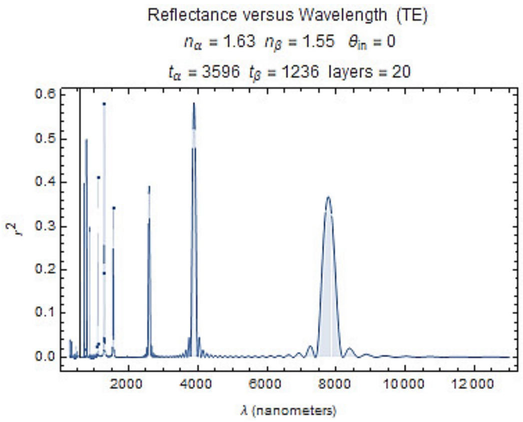
</td>
<td>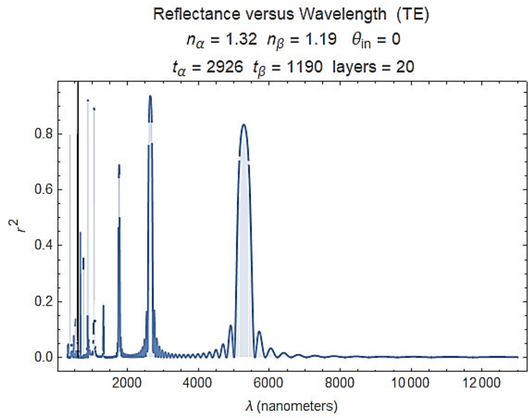
</td>
</tr>
</table>

Publisher's Note: MDPI stays neutral with regard to jurisdictional claims in published maps and institutional affiliations.

© 2020 by the authors. Licensee MDPI, Basel, Switzerland. This article is an open access article distributed under the terms and conditions of the Creative Commons Attribution (CC BY) license (http://creativecommons.org/licenses/by/4.0/).
